# Digital haptics improve speed of visual search performance in a dual-task setting

**DOI:** 10.1038/s41598-022-13827-5

**Published:** 2022-06-16

**Authors:** Ruxandra I. Tivadar, Rebecca C. Arnold, Nora Turoman, Jean-François Knebel, Micah M. Murray

**Affiliations:** 1 The LINE (Laboratory for Investigative Neurophysiology), Department of Radiology, Lausanne University Hospital and University of Lausanne, Lausanne, Switzerland; 2 Department of Ophthalmology, Fondation Asile des Aveugles and University of Lausanne, Lausanne, Switzerland; 3 Cognitive Computational Neuroscience Group, Institute of Computer Science, University of Bern, Bern, Switzerland; 4 Working Memory, Cognition and Development Lab, Department of Psychology and Educational Sciences, University of Geneva, Geneva, Switzerland; 5 CIBM Center for Biomedical Imaging, Lausanne, Switzerland; 6 The Sense Innovation and Research Center, Lausanne and Sion, Switzerland

**Keywords:** Attention, Perception, Cognitive neuroscience, Sensory processing

## Abstract

Dashboard-mounted touchscreen tablets are now common in vehicles. Screen/phone use in cars likely shifts drivers’ attention away from the road and contributes to risk of accidents. Nevertheless, vision is subject to multisensory influences from other senses. Haptics may help maintain or even increase visual attention to the road, while still allowing for reliable dashboard control. Here, we provide a proof-of-concept for the effectiveness of digital haptic technologies (hereafter digital haptics), which use ultrasonic vibrations on a tablet screen to render haptic perceptions. Healthy human participants (N = 25) completed a divided-attention paradigm. The primary task was a centrally-presented visual conjunction search task, and the secondary task entailed control of laterally-presented sliders on the tablet. Sliders were presented visually, haptically, or visuo-haptically and were vertical, horizontal or circular. We reasoned that the primary task would be performed best when the secondary task was haptic-only. Reaction times (RTs) on the visual search task were fastest when the tablet task was haptic-only. This was not due to a speed-accuracy trade-off; there was no evidence for modulation of VST accuracy according to modality of the tablet task. These results provide the first quantitative support for introducing digital haptics into vehicle and similar contexts.

## Introduction

The 2018 World Health Organization (WHO) global status report on road safety highlights that the number of annual road traffic deaths has reached 1.35 million^1^. Road traffic injuries are now the leading killer of people aged 5–29 years. Driving makes intense demands on visual perception^2^. Multiple resource theory suggests that two tasks that draw upon the same sensory modality, code (i.e., analogue/spatial vs. categorical/verbal processes), or stage of processing, will interfere with each other more than two tasks that draw upon different resources^3,4^. Introducing in-vehicle tasks with a significant visual component can overload drivers, as time spent looking inside the vehicle is not spent looking at the road for potential hazards^5,6^. Cognitive load can also delay or interrupt cognitive processing of roadway-related information, resulting in longer reaction times^7,8^. As a result, operating devices that require glances away from the road can lead to structural interference, which can have negative effects on driving performance^9–11^, inducing a type of visual impairment for the road. Increasing the duration of glances away from the road increases the probability of lane departure, such that glances of 2 s lead to 3.6 times more lane departures than do glances of 1 s^5^. Thus, as drivers increase their use of in-vehicle devices there is an associated increase in related crashes^12^. Recent technological innovation in vehicles has included the implementation of touch screen tablets into the dashboard of a car. The use of a tablet in a car likely shifts the visual attention of a driver away from the road towards the screen. Visual attention during driving is an important predictor of accident involvement^11,13,14^.

Over the past 20 or so years, it has become increasingly recognised that visual functions operate within a multisensory framework, whereby other sensory modalities impact visual perception and attention throughout their processing^15–18^. With regards to spatial attention, haptic information can successfully complement or even replace visual information^19,20^. The haptic system is a perceptual system that is active during tactile exploration. Spatial object characteristics (i.e. shape, topology, location in space) in particular, can be supported by visual, tactile, and auditory information (reviewed in^21^). Spatial object representations pertaining to object shape or topology, and to an object’s location in a certain space or with regards to other objects, can be achieved in a largely modality-independent fashion^22^, and engage a common representational coordinate system^23,24^. To minimize visual overload, the haptic system can thus be used to support spatial representations of objects, bypassing the visual system. A particularly striking example is in the case of driving simulation or similar scenarios, where tactile cues have been shown to be effective as warning signals^25–29^ and to enhance visual attention^25^. To date, however, the use of haptics to control interfaces while maintaining driver performance has not been investigated.

Technological innovations have created new methods for digitization of information, such as tablets digitally rendering tactile information (e.g. Xplore Touch from Hap2U; http://www.hap2u.net), which we hereafter refer to as a ‘haptic tablet’. This type of haptic tablet uses ultrasonic vibrations to modulate the friction of a flat screen under the actively exploring finger, and thus simulates the localized perception of texture^30,31^. Such devices are proving to be especially effective as sensory substitution devices for the visually-impaired, given their ability to simulate the perception of objects and their very low training requirements^32,33^. What is distinct about these technical innovations is their ability to render recognisable and manipulable textures and objects, rather than simply provide localized haptic feedback. These advantages might prove helpful when applied to driving environments (see e.g.^34^ for discussion).

The digital nature of novel haptic devices surpasses limitations that classical devices suffer from, such as a pre-defined library of stimuli. For example, digital tablets can offer a much more complex dashboard, including controls of many in-car functions, while a physical dashboard remains limited by the available physical space in the vehicle. In fact, digital innovation of mobility services is expected to fully change the automotive ecosystem, resulting in improved driver safety, well-being, comfort, and entertainment, as well as improved vehicle management and autonomy^35^. However, the vast majority of extant haptic technologies in touch screens still only confer haptic signals regarding finger position on the touch screen^36,37^ rather than impressions of interaction or control over an interface itself or emulation of a button or slider (discussed in Ref.^34^).

Here, we investigated the effectiveness of haptic feedback on a secondary and concurrent task to protect or augment performance on a primary and centrally-performed visual search task. To this end, we tested participants as they simultaneously completed two asynchronous tasks: a centrally presented visual conjunction search task (primary task) and a laterally presented control of sliders on a tablet (secondary task intended to mimic controls that might appear on a car dashboard). We tested three different styles of sliders (horizontal, vertical, and circular) under different modalities: visual-only, haptic-only, and visual-haptic (i.e. multisensory). We expected participants to show improved performance on the primary visual conjunction search task when the secondary tablet task was performed under haptic-only conditions, presumably by alleviating divided visual attention to both tasks. We had no strong a priori hypothesis regarding whether this improved performance would manifest in faster reaction times (RTs) and/or more accurate task completion. Similarly, and because of the general dominance of visual processing in sighted adults, we also expected performance on the secondary, tablet task to be highest under visual-only and multisensory conditions.

## Results

**Figure 1:**
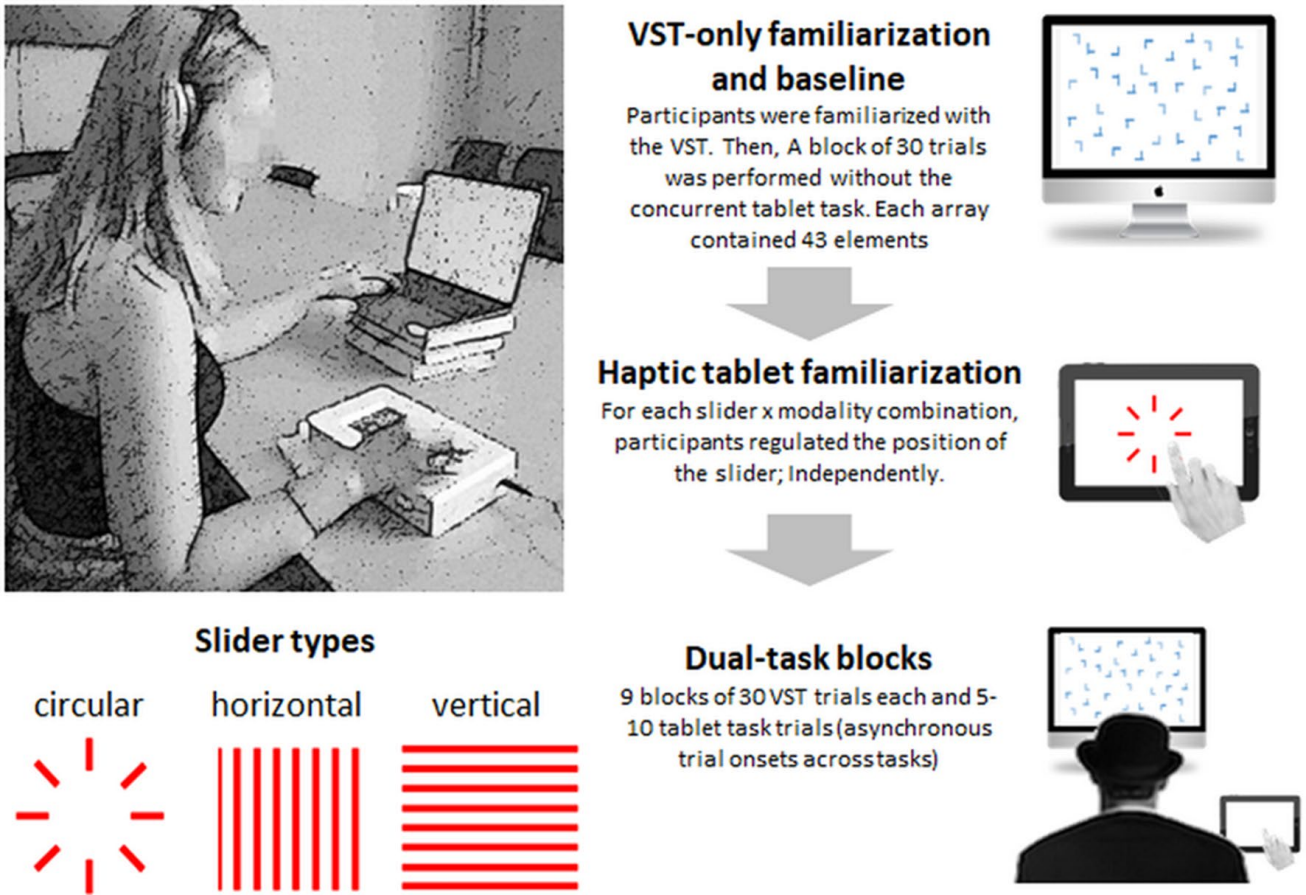
Overview and synopsis of the experimental paradigm. Participants simultaneously performed the central visual search task (VST) as well as a task on the haptic tablet that involved control of vertical, horizontal or circular sliders. These sliders were in turn presented either in a purely unisensory haptic, unisensory visual, or multisensory manner. The right side of the figure illustrates the sequence of familiarization and tasks performed by the participants.

Participants were required to complete a colour × shape feature conjunction visual search task (VST) while interacting with the haptic tablet (Fig. 1). In the VST, participants were exposed to 15 target-present layouts and 15 target-absent layouts (30 trials per block). Each layout was comprised of an array of 43 stimuli (25 light blue L letters, and 18 dark blue T-like letters). In target present trials, one of the T-like letters was light blue (i.e. the target). In the tablet task, participants were required to regulate the slider. The form of the slider and the sensory modality of the information available (i.e. visual, haptic, and multisensory) were held constant during a given block of trials. First, participants completed a baseline VST measurement (i.e. a block of 30 trials performed without the tablet task). Performance on this block of trials resulted in group-averaged (± s.e.m.) RTs of 3.25 ± 0.07 s, accuracy of 73 ± 3%, false alarms on 12 ± 2% of trials, and misses on 5 ± 1% of trials. These data provide an index of the relative difficulty of the VST at the onset of the experiment.

Subsequently, blocks of VST trials were combined with each slider and modality type across subsequent 9 blocks of trials; the ordering of which was randomised across participants. In total, participants completed 270 trials of the VST (9 conditions × 30 stimuli, excluding the abovementioned 30 baseline trials). The number of trials on the tablet task ranged from 5 to 10 on any block of trials (median of 7) and depended on participant’s performance as the duration of a block of trials was anchored to the VST (see “Methods” section).

**Figure 2:**
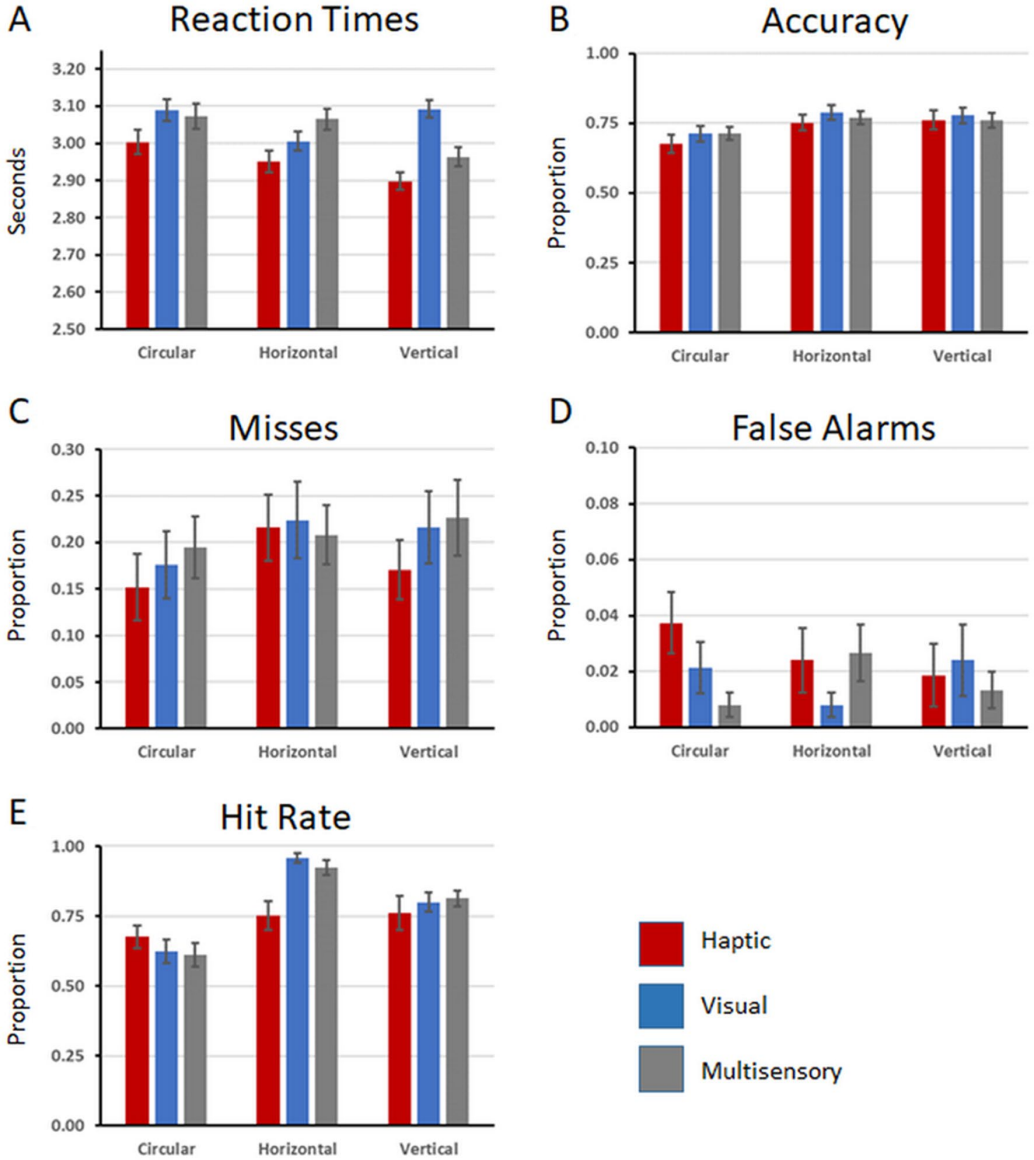
Group-averaged (N = 25) behavioural results from the VST (**A–D**) and from the tablet task (**E**). (**A**) Reaction times on the VST measured in seconds. (**B**) Accuracy on the VST measured as a proportion. (**C**) Misses on the VST measured as a proportion. (**D**) False Alarms on the VST measured as a proportion. (**E**) Hit rates on the tablet task measured as a proportion. Across all of the panels, the error bars indicate the standard error of the mean.

A synopsis of the results is provided in Table 1, and the data from the full 3 × 3 design are illustrated in Fig. 1. For RTs during the VST, the 3 × 3 rmANOVA revealed a main effect of Modality (F_(2,48)_ = 7.146; p = 0.002; η_p_^2^ = 0.229). No main effect of Slider nor the interaction between factors were observed (F’s < 2). Shapiro–Wilk tests indicated that the residuals were normally distributed. After collapsing across slider types, post-hoc contrasts (Holm–Bonferroni corrected) revealed that RTs on the VST were faster when the tablet task was performed with the haptic vs. visual modality (mean ± s.e.m.: 2.95 ± 0.05 vs. 3.06 ± 0.05 s; t_(24)_ = 4.25; p < 0.001) as well as with the haptic vs. multisensory modality (2.95 ± 0.05 vs. 3.03 ± 0.04 s; t_(24)_ = 2.60; p = 0.016), while the VST RTs when the tablet task was performed in the visual or multisensory modalities did not significantly differ (t_(24)_ = 0.88; p > 0.38) (Fig. 2A).

**Table 1:** Summary of results of 3 × 3 rmANOVAs and post-hoc tests.

	Significant main effect of modality	Significant main effect of slider	Significant modality × slider interaction
RTs (VST)	Yes VST completion faster when tablet task was haptic than either visual or multisensory	No	No
Accuracy (VST)	No	Yes VST accuracy worse when the tablet sliders were circular than either horizontal or vertical	No
Misses (VST)	No	Yes Misses on the VST were fewer with circular than either horizontal or vertical sliders	No
False alarms (VST)	No	No	No
Hit rate (Tablet)	Yes	Yes	Yes

For accuracy during the VST, the 3 × 3 rmANOVA revealed a main effect of Slider (F_(2,48)_ = 13.324; p < 0.001; η_p_^2^ = 0.357). Neither the main effect of Modality nor the interaction were significant (F’s < 2). The Shapiro–Wilk test indicated that the residuals were not normally distributed. Consequently, a permutation-based ANOVA was conducted. This analysis revealed a main effect of Slider (p < 0.001), but no main effect of Modality nor interaction between factors. After collapsing across modalities, post-hoc non-parametric contrasts indicated that the main effect of Slider was explained by generally poorer performance on the VST when the slider for the tablet task was circular (70 ± 3%) than either horizontal (77 ± 2%; Z = 3.178; p < 0.001) or vertical (77 ± 3%; Z = 3.633; p < 0.001); the latter two of which did not significantly differ (Z = 0.272; p > 0.78) (Fig. 2B).

The 3 × 3 rmANOVA with the rate of misses on the VST revealed a main effect of Slider (F_(2,48)_ = 4.063; p = 0.023; η_p_^2^ = 0.145), but no main effect of Modality nor any significant interaction (F’s < 2). The Shapiro–Wilk test indicated that the residuals were not normally distributed. Consequently, a permutation-based ANOVA was conducted. This analysis revealed a main effect of Slider (p = 0.01), but no main effect of Modality nor interaction between factors. After collapsing across modalities, post-hoc contrasts indicated that the main effect of Slider was explained by generally fewer misses on the VST when the tablet task was performed with the circular slider (17 ± 3%) than with either the horizontal (22 ± 3%; Z = 2.395; p = 0.017) or vertical sliders (20 ± 3%; Z = 2.019; p = 0.044); the latter two of which did not significantly differ (Z = 0.798; p > 0.425) (Fig. 2C). The 3 × 3 rmANOVA with the rate of false alarms on the VST revealed neither a main effect nor the interaction between the factors of Modality and Slider (all p’s > 0.1).

The analysis of hit rates on the tablet task, which entailed the 3 × 3 rmANOVA, revealed main effects of Modality (F_(1.347,32.337)_ = 141.71; p < 0.001; η_p_^2^ = 0.855) and Slider (F_(2,48)_ = 16.868; p < 0.001; η_p_^2^ = 0.413) as well as a significant interaction between these factors (F_(2,48)_ = 8.247; p < 0.001; η_p_^2^ = 0.256) (see Fig. 1E). The Shapiro–Wilk test indicated that the residuals were not normally distributed. Consequently, a permutation-based ANOVA was conducted. This analysis revealed a main effect of Slider (p < 0.001), a main effect of Modality (p < 0.001), as well as an interaction between factors (p < 0.005).

In terms of the main effect of Modality, performance on the tablet task was generally worse when completed using haptics alone (32 ± 4%) versus either visual alone (79 ± 2%; Z = 4.374; p < 0.001) or with multisensory information (78 ± 2%; Z = 4.374; p < 0.001); the latter two of which did not differ (Z = 0.150; p > 0.88). In terms of the main effect of Slider, performance on the tablet task was generally worse when completed using the circular slider (51 ± 3%) versus either the horizontal slider (73 ± 3%; Z = 3.915; p < 0.001) or vertical slider (65 ± 3%; Z = 2.813; p < 0.005); the latter two of which also significantly differed (t_(24)_ = 2.161; p = 0.031). Given the significant interaction additional non-parametric Friedman tests were conducted for each modality separately. When the task was performed haptically, there was no reliable effect of slider (Χ^2^_(2, N=25)_ = 0.881; p = 0.644). When the task was performed visually, there was a reliable effect of slider (Χ^2^_(2, N=25)_ = 26.674; p < 0.001). When the task was performed with multisensory information, there was a reliable effect of slider (Χ^2^_(2, N=25)_ = 27.830; p < 0.001). In both cases, performance on the tablet task was best when the slider was horizontal, followed by vertical that was better than circular (all p’s < 0.015).

We also examined whether there was evidence of a trade-off between the VST and tablet tasks by testing for correlations in accuracy for each the nine modality × slider pairings (using Kendall’s tau-b). There was no evidence for any significant correlations (all p’s > 0.09). Moreover, instances of non-significant trends followed from positive correlation coefficients, suggestive against a trade-off account wherein negative coefficients would be expected.

## Discussion

These results provide the first quantitative support for introducing digital haptics into automobile tablet interfaces. After only fifteen minutes of task familiarisation, participants were able to successfully perform a tablet task requiring the control of sliders, providing an indication of their ability to interface with the technology. Following this brief period of task familiarisation, participants were confronted with the task of controlling the sliders on the haptic tablet while simultaneously completing a central visual search task. Visual search performance was fastest when participants simultaneously performed the tablet task with only haptic information available. A simple speed-accuracy trade-off could not account for this RT advantage, because accuracy did not reliably differ on the visual search task as a function of the modality of the tablet task. The modality of the tablet task impacted RTs, but not accuracy, misses, nor false alarm rates on the VST. There is no evidence from this pattern of results for a deleterious effect of introducing digital haptics into this dual-task scenario (see also^38,39^).

Performance on the tablet task was lower for the haptic-only condition compared to conditions in which visual information was available. This was not altogether surprising, given that the task required participants to adjust the slider to a specific position that can more readily be checked and adjusted based on visual feedback (when present). As digital haptic technology is relatively new, and thus participants are not accustomed to the sensation of texture mediated through ultrasonic vibration, we anticipated that performance with the technology would not be maximal, particularly in a demanding dual-task setting. Nonetheless, participants successfully interacted with the tablet after only 15 min of familiarisation, completing our baseline requirement. It is moreover important to point out that the visual task was designed to have a high attentional load with 43 items in a display and was also performed with a time limit of 5 s per trial. More extensive training would likely improve performance on the tablet task under haptic-only conditions. In fact, previous results show that after a training session of about 45 min on the haptic tablet, blindfolded sighted participants as well as visually impaired individuals were able to discriminate letters and to build mental representations of these letters that they then mentally manipulated in a mental rotation task^32,33^.

Performance on the VST varied according to the slider type. The circular slider led to the least accurate performance on the VST for all modalities, but also the fewest missed trials. Moreover, circular sliders also led to the lowest hit rate (independent of modality) on the tablet task. Practical and design constraints led to the circular slider having the least amount of available texture on the tablet screen, as lines in the circular button were shortest. Despite our efforts to maintain a similar central presentation of all three sliders on the screen of the tablet, as well as similar proportions and similarity to existing digital slider button types, it is possible that elongating the bars and uniting them at the centre would have resulted in better performance, and not in confusion, as the different bars would have been felt when the circle would have subtended smaller sizes as well. However, this would have introduced some type of bias towards the more “adaptable” circular button, which we wanted to avoid. In addition, the limits of haptic shape processing and the way in which different textures guide human interaction and perception are currently largely unknown. Therefore, future research endeavours, that our laboratory as well as other teams are currently undertaking, should focus on studying this aspect in more depth. However, we would caution against a priori dismissal of circular sliders in haptic tablet interfaces for several reasons. First, as noted about this type of slider, it resulted in the fewest missed trials on the VST. Second, it remains unknown how manipulation of such sliders could be trained or otherwise improved by including more finger and/or gesture-based control. For example, limitations of the tablet technology used here are that it required active exploration (i.e. the texture is felt only when the finger is moving along the tablet surface) as well as that it was limited to single-digit use. These will undoubtedly be surmounted with continued technological developments.

The present results demonstrate the beneficial and alleviating effect that haptic feedback can exert on visual functions in situations in which vision is effectively impaired; here participants were dividing their visual attention between two tasks at two locations; a situation that can be considered a proxy for a driving task. Looking away from the road while driving in order to interact with in-car technologies can result in visual interference and, as a result, in impaired driving performance^9^. In-car devices offer a wide variety of visual information^40^, and thus compete with driving tasks over limited visual resources^41^. In fact, so far, visual attention performance seems to be the most important predictor of accident involvement, as about 25–37% of crashes involve some form of driver inattention^42^. Even applications that do not require glances away from the road, such as speech recognition systems, or hands-free cell phone use^28,43,44^ can nevertheless impose a cognitive load that may interfere with driving performance^9^. This cognitive load has the potential to impair drivers’ ability to maintain vehicle control^45^. In addition, aging^11^, brain injury^46^, stroke^47^, or other syndromes such as Alzheimer’s or Parkinson’s disease^48,49^ can severely impair visual attention performance. Neuropsychological tests that require visual perception and visual spatial judgments are the most useful screening measures for hazardous driving in such populations^49^. An example of such a test is given by the Useful Field of View (UFOV)^50^, which tests aspects such as processing speed, divided attention, and selective attention. This suggests that divided attention is one of the important indicators of driving performance, validating our choice of task as an approximation of a driving-like implementation of our attentional function.

Focalized training of visual attention, for example through video games^51^ or simulator training^52^, or even directly on UFOV tasks^53^ can significantly improve UFOV scores^51,53^. Given the multisensory nature of our brain^16,18,54,55^, recent efforts to improve driver visual attention focus on auditory and haptic stimulation to alleviate the visual load of the drivers, with very effective results^25–29^. Our results support such applications, by showing that in a divided attention situation in a dual-task setting, the use of a haptic-only rendering of a button in a secondary task significantly facilitated the speed of successful performance on the primary visual task (here the VST). As our difficult VST was meant to simulate visual processes similar to those active during driving situations, such as selective and visuo-spatial attention, these results indicate that simulated haptic feedback is apt at alleviating the visual load and at improving visual attention performance.

As exemplified above, multi-tasking in a driving situation can effectively result in ‘impaired’ visual processing of the visual scene (i.e. the view through the windshield and the road in particular). This is exacerbated by dashboard technologies based on visual displays instead of physical (and therefore haptically manipulable) buttons. When we try to imagine a physical dashboard that includes various functions, we can only implement a certain amount of buttons until the car becomes non-ergonomic. With increasing number and complexity of controls and information displays in the car, international standardization agencies have started to standardize the appearance, arrangement, and reachability of operational controls, indicator units, and control lamps in the vehicle^56^. The aim of such standards is mainly to enhance driving experience and road safety, as well as to improve operation efficiency by optimizing and harmonizing the arrangement and placement of indicator systems and control elements^56,57^. Car owners spend a large portion of their daily time in cars^58^. Individual needs and desires of each user can vary significantly, and they must not be neglected by functionality providers. To meet the needs of individual users, car manufacturers and software and hardware developers are implementing an increasing number of functions and technologies into new-generation cars^58,59^. This is only possible using digital technologies, as the physical space of a car is restrained. Within the automotive industry, digitization shifts the technological focus from physical to digital, enables customers to bring in their changing understanding of mobility, and makes them an ever more valuable source of information^60^, driving developments even further. Our results add to these developments, in that they support the implementation of a new technology, that not only promises to improve in-car ergonomics, but also to increase safety and allow a wider range of functions to be implemented in a car dashboard, thereby improving user satisfaction and personalisation. As such, and although digital haptic technologies are at their very first beginnings, they are a promising contribution to the ever-developing automotive industry.

In conclusion, our results support the implementation of digital haptics in automobiles and other vehicles, by indicating that they help alleviate overloaded visual functions by focusing visual attention on a primary, central task that we here took as a proxy for driving performance. While more experience/practice should reasonably improve user interaction with the simulated haptic objects, we demonstrate that such technologies do not require an extensive and exhaustive familiarisation process. As such, digital haptic technologies promise to improve driving efficiency as well as users’ comfort with minimal accommodation efforts. Research in this field is at its very beginning, and digital haptic technologies are quickly evolving. For this reason, we are confident in the fast development in this field that will lead to a growing implementation of these technologies not only in the automotive industry, but also in human–computer interfaces more generally.

## Methods

### Participants

All participants provided written informed consent to procedures approved by the Vaudois cantonal ethics committee (protocol 2018-00240). All procedures have been performed in accordance with the Declaration of Helsinki. We tested 25 adults (18 women and 7 men, age range 20–27 years, mean ± stdev: 21.56 ± 1.66 years), who volunteered for our experiment. Participants reported normal or corrected-to-normal vision. All our participants self-reported to be right-handed. We also asked our participants about their experience with video games, due to previous evidence of improved selective attention in players^51,61^. Only five participants reported playing video games (mean ± stdev: 8.5 ± 12.35 h a week). The remaining twenty participants did not report playing any video games. We also measured the length and width of the distal phalanx of right index finger in our participants (average length ± stdev: 2.63 ± 0.291 cm, average width ± stdev: 1.73 ± 0.152 cm), in case failures to detect the bars could be related to different finger sizes in our participants. However, throughout the task familiarisation period, they all successfully managed to feel the slider button bars.

### Apparatus

Haptic stimulation was delivered via a tablet with a TFT capacitive 7-inch touchscreen with a resolution of 1024 × 600 pixels. The screen of the tablet is controlled by a Raspberry Pi 3 based system, and the operating system is Raspbian (Linux). The processor of the tablet is a Broadcom ARMv7, quadcore 1.2 GHz and it has 1 Go RAM and Rev C WaveShare. The tablet comes with a haptic creation tool, which is a software that allows for user control of haptic textures. Several other APIs based on JavaScript are installed, such as library tools that allow the implementation of haptics on other applications. Figures in jpeg format were re-coded in haptic format using a kit written in C++. For more technical details describing the rendering of the haptic feedback, see^30,31,62^. The conjunction search task was run on a 13-inch Mac computer (Retina, 13-inch display, 2018).

### Stimuli

**Figure 3:**
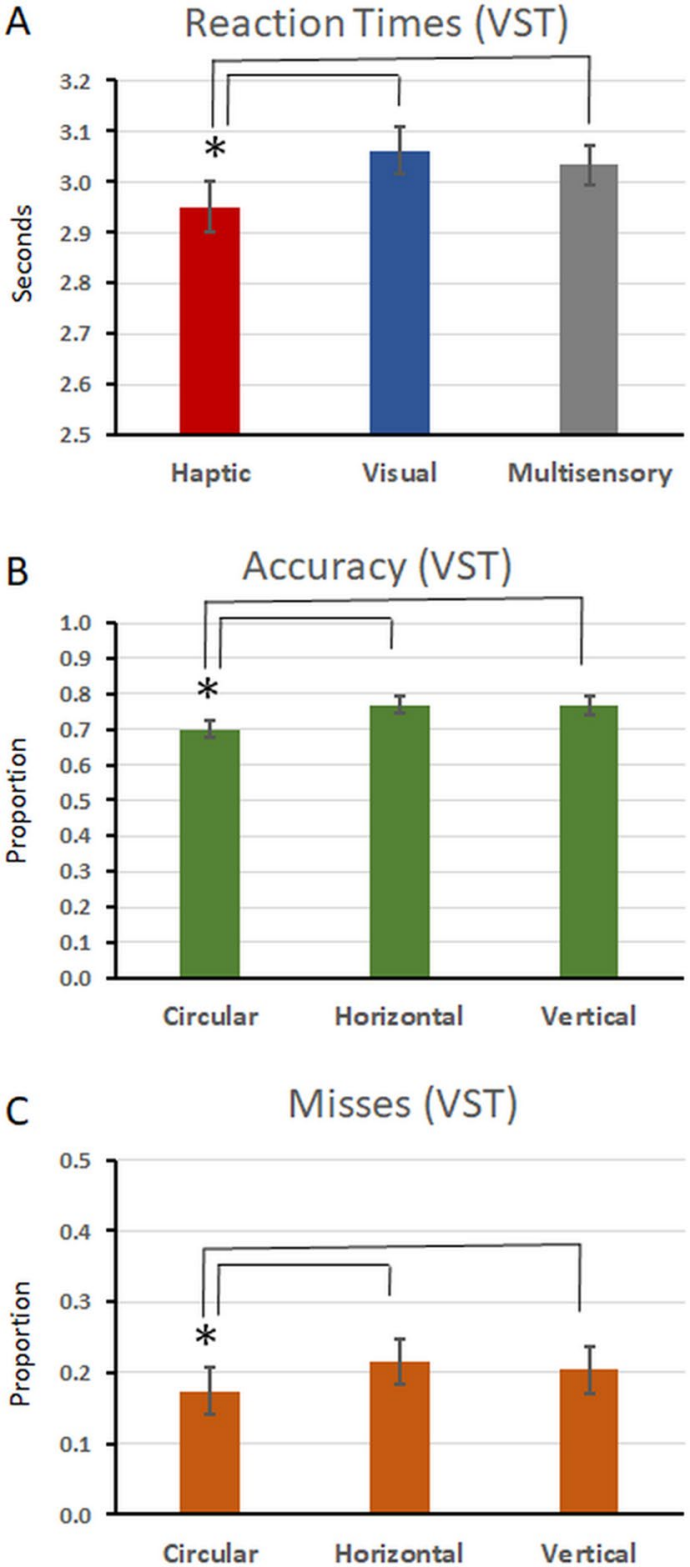
Group-averaged (N = 25) data from the VST task. (**A**) Displays the RT data collapsed across sliders. (**B**) Displays the accuracy data collapsed across modalities. (**C**) Displays the misses collapsed across modalities. In all panels, the error bars indicate the standard error of the mean. Asterisks indicate a significant difference (p < 0.05; Holm–Bonferroni corrected).

Tablet stimuli consisted of three slider buttons—a horizontal one, a vertical one, and a circular one (see Fig. 3). The sliders were created to always appear centrally on the screen of the haptic tablet (1024 × 600 px), and to respect the same proportions. The width of the bars of the vertical slider button was 27 px (0.7 cm), their length 463 px (12.25 cm), and the spaces between bars subtended 50 px (1.3 cm). The width of the bars of the horizontal button was 27 px, while their length was 714 px (18.89 cm), and spaces subtended 50 px (1.3 cm). The bars on the circular slider had a width of 27px, and a length of 131 px (3.47 cm), with spaces between them of 120 px (3.18 cm). The length of the bars on the circular slider were shorter than the bars used for the horizontal or vertical buttons, in order to create a button that would better resemble other circular slider buttons on the market, which would ease user interaction. If the bars would have been longer, and thus connected in the middle (i.e. a star-like button), this could have induced the participants into error. Specifically, if the circular bars would have been united in the middle, it would have been easy to misunderstand the bar position, as the exploration on the tablet is dynamic and happens in vertical sweeps. The spaces between bars of the circular slider were wider, due to the circular clock-like arrangement of the bars and their shorter length. Stimulus size for all three sliders was 1024 × 576 px. Sliders, when represented as an image, appeared centrally on a white background. White pixels did not result in a texture on the finger. All non-white pixels were coded with the same haptic texture, which was created using the Hap2u pre-installed Texture Editor software. The ultrasonic vibration was adjusted to have a right triangular shape, as this offers the most intense and quick reduction of the friction of the screen under the finger, thus conferring a rather sharp and pointy sensation. In contrast, a sinusoidal-shaped wave would confer a rather smooth sensation, which would be more difficult to detect by the user. The period of the window of one triangular ultrasonic signal was chosen to be 1290 µm (which is considered a “coarse” texture, see^63,64^ and the amplitude was set at 100%, corresponding to roughly 4 µm^30^. The inter-stimulus interval varied randomly between 1 and 8 s.

Stimuli for the conjunction search task^65–67^ were created in Microsoft Paint. In total, 30 different layouts were created for the visual search task (VST), out of which 15 were target-present layouts and 15 were target-absent layouts. Each layout was comprised of 43 stimuli, of which 25 were light blue L letters, and 18 were dark blue T-like letters in target absent trials. In target present trials one of the T-like letters was light blue (i.e. the target). These T-like stimuli were a spatial feature combination of the L and the T letter, that were designed in this way in order to make the task harder, as it is known that each dimension contributes additively to conjunction search rates^68^ (see Fig. 3). The layouts subtended the full screen of the MacBook Air. Before every layout appeared, a blue fixation point was presented in the centre of the screen for 0.5–0.9 s. The 30 layouts within a block of trials were presented in a fully randomized order.

### Procedure and task

We developed a divided-attention paradigm through the combination of a visual attention task and a tablet task to mimic the experience of a tablet-equipped car. The participants wore noise-cancelling headphones (Bose QuietComfort 2) throughout the duration of both the familiarisation period and the experiment. This was to ensure that they did not hear the ultrasound vibration noise of the tablet. The initial task familiarisation period consisted of exposing the participants to each of the tasks independently. On the VST, they completed a 30-trial block. On the tablet, participants were asked to reach a baseline of two correct responses in a row for each of the 9 experimental conditions presented in randomized order (i.e. 3 slider type × 3 modality combination; see below). When this threshold was reached, the experimental phase began. All our participants easily achieved this threshold. The VST and the tablet task were conducted in the same manner as during the familiarisation phase. For the VST, participants scanned 30 randomized layouts of dark-blue T’s and light-blue L’s on a computer screen to determine whether or not a light-blue T was present. The computer was placed with the centre of the screen at eye-level at about 60 cm away from the participant. The participants used their index and middle finger of their left hand to press the ‘x’ key on the keyboard if the light-blue T was present, and the ‘z’ key on the keyboard if the light-blue T was absent. Participants had 5 s to respond before a fixation dot appeared, automatically followed by the appearance of the next layout.

The tablet task was conducted on a Hap2U tablet. This task consisted of three different types of sliders (vertical, horizontal, and circular) as well as three different modalities in which these sliders were presented (visual, haptic, and combined visual-haptic). Overall, a total of nine conditions (i.e. 3 × 3 factorial combination of the slider types and modalities: vertical × visual; vertical × haptic; vertical × visual-haptic; horizontal × visual; horizontal × haptic; horizontal × visual-haptic; circular × visual; circular × haptic; circular × visual-haptic) were tested for each participant. The order of the nine conditions was grouped by modality, and both order of slider and modality were randomized. During this task, participants were asked to slide their right index finger to one of eight different positions on the slider in a randomized order. The target position number (position one through position eight) was stated by the researcher (R.C.A.), and the participants then used their right index finger to move to the correct position on the tablet. Once the participant reached the target position, they were instructed to keep their finger on the tablet until the researcher said “good”. Participants had 10 s to respond before the next slider position was presented. Participants simultaneously completed the two aforementioned tasks by using their left hand for the VST and their right hand for the tablet task (270 trials in total for the double task condition, 30 trials per condition and an additional 30 VST trials as a baseline, without a haptic task). Participants were instructed to complete both tasks to the best of their abilities (i.e. as quickly and accurately as possible). The total time it took the participants to complete the VST for each slider condition was recorded. Once the participant reached the end of the thirty layouts on the VST, the program finished and they had completed the condition. Reaction times and accuracy were measured throughout the task. The tablet task was also ended at this point, and the number of successful trials was recorded. After all nine blocks of trials had been completed, the participant had concluded the experiment. Stimulus delivery and behavioural response collection were controlled by Psychopy software^69^ for both the VST and the tablet task.

### Data analysis

Performance on the VST was evaluated using: (1) reaction times to hits, (2) accuracy rates (i.e. the percentage of hits and correct rejections), (3) the percentage of false alarms, and (4) the percentage of misses. Performance on the tablet task was evaluated using the percentage of correct trials. Data were pre-processed in Matlab and analysed with SPSS version 27 (IBM Inc.). For the analysis of RTs on the VST, we first excluded all missed trials (11.3% of trials), which were trials where a response was not given within 5 s. We then excluded any remaining outlier trials on a single subject basis (i.e. for each subject and condition), applying a mean ± 2 standard deviations criterion to their RTs, which resulted in the exclusion of 3.23% of trials (see^70^). Finally, we selected only those data stemming from correct trials. Data were submitted to 3 × 3 repeated measures ANOVAs with within-subject factors of Modality (haptic, visual, multisensory) and Slider (vertical, horizontal, circular).

For the tablet task, we only recorded hit rates, which were analysed with a 3 × 3 repeated measures permutation ANOVA with within factors Modality (visual, haptic, multisensory) and Slider (vertical, horizontal, circular). The Greenhouse–Geisser correction for non-sphericity was used when appropriate, and adjusted degrees of freedom are reported in such instances. Effect sizes are reported using partial eta-squared (η_p_^2^). Post-hoc analyses were performed with paired t-tests, applying Holm-Bonferroni correction for multiple comparisons^71^. In the event that residuals were not normally distributed, we then conducted permutation based ANOVAs using R (R core team, 2021) and 5000 permutations of the dataset. Unfortunately, technical constraints of the prototype haptic tablet used here did not allow us to record RTs. Specifically, when the screen was “active” in that it could output these data, the touch screen was activated and each of the first touches that participants made to start exploring the haptic display was recorded as a “response”—i.e. a mouse press (we would remind the reader that the technology of digital haptic rendering only works when the person interacting with it dynamically explores the screen). Consequently, we were obliged to switch the active screen off to avoid false responses and thus had not possible way to record unbiased RTs. Accuracy was recorded directly by a single experimenter (R.C.A.) for all participants.

## Data Availability

The datasets generated during and/or analyzed during the current study are available from the corresponding author on reasonable request.
